# Cannabis and Nicotine Substance Use Coercion During the Perinatal Period

**DOI:** 10.1177/26884844251387024

**Published:** 2025-10-08

**Authors:** Peyton Groves, Callie Laubacher, Yesmina Salib, Erin Mickievicz, Virginia Duplessis, Nicole Molinaro, Dara D. Méndez, Katherine Guyon-Harris, Ruben G. Martinez, Judy Chang, Natacha M. De Genna, Maya I. Ragavan

**Affiliations:** ^1^ Division of General Academic Pediatrics, University of Pittsburgh and UPMC Children’s Hospital of Pittsburgh, Pittsburgh, Pennsylvania, USA.; ^2^ Futures Without Violence, San Francisco, California, USA.; ^3^ Women’s Center and Shelter, Pittsburgh, Pennsylvania, USA.; ^4^ Department of Epidemiology, University of Pittsburgh School of Public Health, Pittsburgh, Pennsylvania, USA.; ^5^ Department of Psychiatry and Human Behavior, The Warren Alpert Medical School, Brown University, Providence, Rhode Island, USA.; ^6^ Department of Obstetrics, Gynecology and Reproductive Sciences, University of Pittsburgh, Pittsburgh, Pennsylvania, USA.; ^7^ Departments of Psychiatry and Epidemiology, University of Pittsburgh, Pittsburgh, Pennsylvania, USA.

**Keywords:** cannabis, intimate partner violence, nicotine, perinatal period, pregnancy, postpartum, substance use coercion, tobacco

## Abstract

**Introduction:**

Perinatal intimate partner violence (IPV) and perinatal cannabis and nicotine product use are common and associated with negative maternal–infant health outcomes. Substance use coercion (SUC), which involves abusive partners’ controlling behaviors related to substance use, has not been studied for cannabis and nicotine products. Perinatal individuals may be particularly vulnerable to SUC, and this study aims to explore how cannabis and nicotine SUC manifests among perinatal survivors.

**Methods:**

We conducted virtual, semi-structured, 45-minute retrospective interviews with 19 IPV advocates and 15 perinatal IPV survivors. Participants were recruited through local IPV agencies and an online recruitment repository. Audio-recorded interviews were transcribed and analyzed using a deductive–inductive thematic analysis approach. Two research team members individually coded each transcript and met to resolve discrepancies.

**Results:**

Key themes emerged relating to survivors’ use, interpersonal coercive tactics, and systems-level control. Specific findings included (1) survivors using substances to cope; (2) abusive partners coercing survivors through withholding, controlling, and punishing substance use; (3) shame and stigma are key drivers of coercion; (4) partners hinder cessation efforts; and (5) child protective services and health care are systems used by abusive partners to control and manipulate perinatal survivors of IPV.

**Discussion:**

The findings demonstrate that cannabis and nicotine products are used to coerce and control survivors of IPV. Future work should focus on developing survivor-centered interventions within systems to better support perinatal IPV survivors using cannabis and nicotine products. This study highlights the impact of cannabis and nicotine SUC on perinatal individuals and underscores the need to consider SUC when providing resources or treatment.

## Introduction

The perinatal period is a dangerous time for survivors of intimate partner violence (IPV), with one in four pregnant people experiencing IPV and 50% of homicides and suicides during the prenatal period being related to IPV.^1–3^ Use of cannabis and nicotine products is also common in the perinatal period, impacting around 11% of perinatal people, with even higher prevalence for those experiencing mental health symptoms.^4–6^ When we refer to nicotine, we mean tobacco and vaping use. When we refer to cannabis, we mean smoking and ingesting (*e.g.*, edibles). Perinatal nicotine rates range from 6% to 10%; similarly, about 6% of pregnant people screen positive for cannabis use.^7,8^ Perinatal IPV and perinatal nicotine and cannabis use are both associated with negative maternal–fetal health outcomes (*e.g.*, low birth weight, preterm birth, insufficient prenatal care),^1,5,9,10^ and previous literature has demonstrated strong associations between perinatal experiences of trauma and increased use of cannabis or nicotine.^11,12^ Perinatal IPV survivors who use nicotine and cannabis, therefore, represent an important group to invest resources and support to improve caregiver and infant health.

Extant literature has demonstrated that survivors of perinatal IPV have high rates of substance use, including nicotine products and cannabis.^13,14^ One factor driving this association may be substance use coercion (SUC) or controlling behaviors related to substance use that can develop within relationships. IPV is rooted in power and control, with partners using numerous behaviors to control, manipulate, or coerce IPV survivors.^15,16^ Most of the SUC work has occurred in the context of opioid use disorder. These studies illustrate how survivors used opioids to cope with trauma, and partners discourage help-seeking through using survivors’ fear of legal repercussions.^17,18^ This work also demonstrated how substance use escalates the severity and frequency of abusive behaviors and can prevent survivors from achieving cessation due to sabotage of recovery efforts.

This formative work with people experiencing opioid use disorder has identified important gaps in the literature around understanding SUC. First, cannabis and nicotine are used more frequently than opioids,^17^ and SUC may manifest differently due to varying legal regulations and differences in addictive potential. At the time of this study, recreational cannabis use was legal in 24 U.S. states, with no known laws specific to pregnant or postpartum use.^19^ Health and hospital systems also have varying and inconsistent policies regarding reporting the prenatal use of marijuana to child protective services (CPS).^20^ Second, limited work has considered SUC within the context of the perinatal period, which is critical as perinatal IPV, nicotine, and cannabis use are prevalent,^1^ and pregnancy is a time when people may be highly motivated in their nicotine and cannabis cessation efforts.^4,18^ Perinatal people also experience more stigma and surveillance around cannabis and nicotine use than non-perinatal people due to attitudes around parenting^21^ and increased contact with systems (*e.g.*, health care).^22^

Understanding cannabis and nicotine SUC is critical for systems to better support perinatal IPV survivors in their cessation efforts and improve resource connection to promote thriving for IPV survivors and their infants. Therefore, this study aims to examine: (1) how nicotine and cannabis SUC manifests for perinatal IPV survivors; (2) how abusive partners may hinder cannabis and nicotine cessation efforts; and (3) systems-level experiences around cannabis and nicotine SUC.

## Materials and Methods

We conducted virtual, semi-structured, individual interviews with IPV advocates and survivors to understand how SUC manifests in the perinatal period for people who use cannabis or nicotine products. We included IPV advocates, as they are professionals with advanced training in responding to IPV, and thus have unique insight into survivors’ experiences.^23,24^ We interviewed postpartum survivors so they could share their experiences over the arc of their prenatal, birthing, and postpartum periods. The University of Pittsburgh Institutional Review Board deemed this study to be exempt.

### Participants and recruitment

Eligibility criteria for survivors included: (1) age 18 or older, (2) speaks and understands English, (3) self-reported as experiencing IPV during the perinatal period within the last 4 years, and (4) used cannabis or nicotine products during the perinatal period. We defined the perinatal period as pregnancy to 1-year postpartum, and IPV was defined broadly to include physical, psychological, emotional, sexual, economic, reproductive, or other forms of coercion and control. Eligibility criteria for advocates included: (1) age 18 or older, (2) speaks and understands English; and (3) currently or previously worked as an IPV advocate. IPV advocates were recruited *via* email from local and state-level victims’ services agencies and coalitions. Survivors were recruited through flyers in local shelters (*n* = 6), from a previous research study (*n* = 1), and an online recruitment repository, Pitt + Me, where participants can register to be notified of studies for which they may be eligible (*n* = 8).

### Interview guides

Separate interview guides for survivors and advocates were developed. Questions focused on (1) survivors’ experiences using nicotine or cannabis during pregnancy, (2) abusive partners’ roles in substance use during pregnancy, and (3) how SUC manifested through systems-level interactions (Supplementary Appendix S1).

### Data collection and analysis

Interviews were conducted on Zoom by one of three trained study team members from March 2023 to January 2024. Interviews took approximately 45–60 minutes to complete, occurred in English, and were audio-recorded and transcribed. Prior to commencing the interviews, we confirmed participants were in a private location and obtained verbal consent. Local IPV and substance use resources were provided after the interview. Participants received $50. Interviews were conducted and analyzed until thematic saturation was reached (after 19 advocate interviews and 15 survivor interviews), when no new codes or themes emerged.^25^ Halfway through data collection, initial results were shared with two community collaboratives of IPV advocates and survivors to discuss emerging themes and ideas for the second round of interviews. Examples of suggestions from collaborative members include recruiting advocates with additional training in social work to participate and adding questions related to social system resource utilization.

We used a qualitative descriptive approach to perform thematic analysis on both sets of interviews.^26,27^ De-identified interviews were uploaded to the Dedoose qualitative software. We started our codebook development focusing on the IPV advocates’ interviews, with a list of *a priori* codes. Two study team members independently coded the first 12 advocate transcripts, adding new codes in an iterative fashion using an editing, constant-comparison approach.^26^ The coders met weekly to identify emerging themes, resolve discrepancies, and add inductive codes. Once the codebook for advocates was finalized, the remaining interviews were coded by one team member, with every third transcript being co-coded. Any discrepancies between coding for transcripts double coded were discussed and adjudicated by a third team member. Upon deep reading of several transcripts from survivors of perinatal IPV, the team noted significant overlap in topics and content. Thus, we opted to use the same codebook developed for the IPV advocates, with any new themes being iteratively added as codes and labeled as survivor specific. Once all transcripts had been coded, the research team met twice to consolidate codes into themes.

## Results

Nineteen advocates and 15 survivors participated in this study. Advocate participants were aged 24–53, 90% female identifying, and 73% non-Hispanic White. Survivor participants were all female identifying, aged 20–44, 60% non-Hispanic White, 6% Hispanic, and 33% Black (Table 1). Substance use included using combustible tobacco only (46%), cannabis and combustible tobacco (27%), cannabis only (20%), and nicotine vape only (7%) (Table 1). Note that only one participant used marijuana edibles, and the rest smoked cannabis, which is consistent with use patterns observed in previous studies.^28,29^ The key cross-cutting themes included: (1) IPV survivors used cannabis and nicotine perinatally to help cope with trauma; (2) abusive partners explicitly used substances to control or coerce IPV survivors; (3) abusive partners used shame associated with perinatal substance use to hurt or manipulate IPV survivors; (4) abusive partners enabled undesired substance use and hindered cessation efforts by IPV survivors; and (5) abusive partners use systems-level coercion to harass, threaten, or control perinatal IPV survivors. The following sections describe each of these themes in more detail and provide illustrative quotations (A: advocate and S: survivor; see Table 2).

**Table 1.** Participant Demographics

*n* (%)	Survivors (*n* = 15)	Advocates (*n* = 19)
Gender		
Woman	15 (100%)	17 (90%)
Man	0 (0%)	1 (5%)
Nonbinary	0 (0%)	1 (5%)
Race and ethnicity		
White	9 (60%)	14 (73%)
African American	5 (33%)	1 (5%)
Latino	1 (6%)	2 (10%)
Other	0 (0%)	2 (10%)
Age		
18–24	3 (20%)	1 (5%)
25–34	7 (46%)	9 (47%)
35–44	5 (33%)	5 (26%)
45–54	0 (0%)	4 (21%)
Number of children		
1	5 (33%)	
2	3 (20%)	
3	5 (33%)	
4	1 (5%)	
5+	1 (5%)	
Substance use		
Cannabis only	3 (20%)	
Combustible tobacco only	7 (46%)	
Nicotine vape only	1 (7%)	
Cannabis and combustible tobacco	4 (27%)	
Cannabis and nicotine vape	0 (0%)	
Years as advocate		
1–2		8 (42%)
3–5		3 (16%)
6–9		6 (32%)
10–15		1 (5%)
16+		1 (5%)

**Table 2.** Themes and Additional Representative Quotations

Theme	Quotes
Theme 1: Perinatal IPV survivors used cannabis and nicotine products to help cope with trauma.	“It helped me to relieve stress. If I’d get upset and I’d smoke, I would feel not so stressed out. I knew it was bad that I shouldn’t be smoking ‘cause I know it could harm the baby. I did try to not smoke as much as I did.”—S11
	“I know quite a few of ‘em would start smoking either tobacco or cannabis just to deal with the stress.”—A14
	“It’s an escape. At that moment, even if it’s for like that five to 15 minutes, to feel out of this world—can I say, to feel out of this world, just to get an escape from being hit on or punched on or verbally being abused.”—S1
	“A person is more agreeable when they’re calm. They might feel like, “If I just totally get high, and I’m not even there to respond to what’s happening in the moment, that they’ll just not bother me. If I’m not even there, they won’t bother me.” I saw that with a client that I worked with in a different setting. She would smoke weed all day, every day. Her partner would come home after work. She would try and turn off and avoid him.”—A15
Theme 2: Abusive partners use controlling and coercive tactics related to IPV survivors’ substance use.	“Whenever he would lock me in the garage, he would open up the blind from the outside … he’d sit there and smoke a cigarette and blow the smoke in the window so I could see him. Then whenever he would hide them, he would go outside and just smoke right in front of me and blow it in the window.”—S7
	“Every time she would smoke, he would use it as a reason why he could abuse her.”—A18
	“He would then take my bag of marijuana, and he would hide it from me. Do you know what I mean? He would just take it and hide it. He would hide my cigarettes, or he would break them. He would do things like that all the time.”—S8
Theme 3: Abusive partners use shame associated with perinatal substance use to hurt or manipulate them.	“There was so much judgment and stigma attached to that beginning in that pregnancy. “Well, look how worthless you are, different things along those lines, because you just can’t kick the cigarettes. You must not love this child.”—A9
	“As far as tellin’ people, yeah. There were times when, “That’s not the topic of conversation that I want to have.” I’m not gonna lie. There’s shame and embarrassment that comes with smoking and pregnancy, at least for me there was. I would try to hide that.”—S3
Theme 4: Abusive partners enabled undesired substance use and hindered cessation efforts by IPV survivors through implicit and explicit coercive behaviors.	“The fighting, the stress. She knew how I was when I would get stressed out, that I would chain-smoke. She didn’t care, of course.”—S4
	“He’ll be pickin’ wit’ me and knowin’ that he done smoked all the cigarettes, or he know I need one after he just picked a fight wit’ me or pickin’ on me, and he’ll start smokin’ his and won’t give me one if I ask.”—S11
	“Partners are just very controlling, so they control a lot of aspects of their life. I’ve a lot of clients, their partner controlled their recovery choices, so even if a pregnant or postpartum person wanted to stop these substances, sometimes their partners just wouldn’t let them. Or even if they wanted to quit these things, their partner would purposely trigger them or create an environment where they would have to continue to use or that wasn’t conducive to recovery.”—A8
	“Yeah, when I wanted to cut back, he would use his vape in front of me. He’d be like, “Well, look what you’re missing out on.” He’d get mad ‘cause if I didn’t wanna do it all the time, constantly like him, because sometimes being pregnant, it made me sick to my stomach to use it, but I kept using it because he kept—to me it seemed like he was influencing it on me to keep using it even though I did wanna cut back.”—S12
Theme 5: Abusive partners use system-level coercion to harass, threaten, or control perinatal IPV survivors.	“Definitely, when it comes to custody. It’s, again, that constant threat [to disclose substance use]. Even if there’s no follow-through on it. If someone is threatening you that they’re gonna report you or that they’re gonna be taking you to court or they’re goin’ to call CYS.”—A6
	“If I didn’t do whatever he said or if he would walk in and I would be hittin’ the joint or whatever, he’d say, “Oh, so now you’re high and smokin’ weed without me, and now I’m gonna have to call CYF and tell them that you’re a drug addict and you’re gonna kill the baby.”—S8
	“Or if they already have CYF involvement, sometimes an abusive partner can use that as a form of power and control, so forcing them to fail urine drug screens or tricking them to fail urine drug screens.”—A8
	“It’s something that can definitely be held over their head. Even after the child is born, you have custody and other issues and stuff like that to work out. In the back of your mind, there is always that constant threat of you can’t pass a drug test, or I’m just gonna report that you’re using drugs.”—AV 006
	“He would tell [people and my doctor] that I’m a horrible mom because I’m smoking cigarettes while I’m pregnant, and I didn’t want anyone to even know. I think that made me—I already felt really, really bad about it, anyways. My doctor knew, though. He told her.”—S10

IPV, intimate partner violence.

### Perinatal IPV survivors used cannabis and nicotine products to help cope with trauma

Participants shared how cannabis and nicotine products were used to cope with trauma, noting that substance use helped to reduce stress, provide a sense of control, and escape from the abuse:

“For pregnant survivors… cigarettes are a huge stress relief … They’ve said, ‘I’m just gonna go have a cigarette,’ when they’re upset.” (A5)

A survivor also shared how substances can provide a sense of control:

“It was the one thing that I could control. I didn’t have control over how my body was startin’ to look and feel. I didn’t have control over how my partner was gonna act, and what was gonna transpire in the relationship. I could control puttin’ a cigarette in my mouth.” (S3)

Other participants talked about how smoking offered an outlet to get away from an abusive situation or get a moment to themselves. An advocate shared: “There was one client, and she was pregnant, and she would go outside to smoke to get breaks from him.” (A18) Survivors also reported using substances, especially cannabis, to mentally escape from abuse: “With the marijuana, it was more like an escaping thing. I’m gonna try to smoke it and get myself so high that I could just block him completely out.” (S8)

### Abusive partners explicitly used substances to control or coerce IPV survivors

Participants described how abusive partners used substances to coerce survivors’ behaviors, such as staying in the relationship or obeying an abusive partner’s wishes, by withholding the cannabis or nicotine as punishment and engaging in physical and emotional abuse as a response to the survivors’ substance use. A survivor noted how her partner would act during fights: “She would withhold my cigarettes when we would fight. She would break them. She would try to make me smoke cigarettes that she knows that I didn’t like… Real manipulative ways like that.” (S4) An advocate shared a similar experience: “Depending on the relationship, they might withhold that from them. They’ll say—specifically, let’s talk about cigarettes—someone might be like, ‘Well, you didn’t do this for me, so I’m not buying you cigarettes today.’” (A18)

Another survivor shared an experience where their substance use was used as a reason to become physically or emotionally abusive: “When he didn’t approve of me smoking while I was pregnant with her, he would try to choke me. He pushed me down the stairs one time. He hit my belly a couple times because I was smoking.” (S7)

### Abusive partners used shame associated with perinatal substance use to hurt or manipulate IPV survivors

Participants noted how abusive partners would use shame around perinatal substance use as a form of emotional abuse and manipulation. A survivor shared: “He’d tell me if I wasn’t with him that nobody would have me… ‘when they find out that you were smokin’ weed while you were pregnant with the baby, they’re gonna think you’re a piece of s***.’” (S8) An advocate noted:

“There could be the shame of you smoking while you’re pregnant, you’re a bad mother, bad birth carrier, birth giver, things like that. I know that there are a lot of abusers that use the fact that the victim smokes or uses marijuana as a way to continue to say, ‘Well, you’re not perfect. You don’t deserve these things because you do X, Y, and Z.’ It can be used as a justification for the abuse, in a way.” (A13)

The stigma associated with perinatal tobacco or cannabis use is also utilized by abusive partners outside of the relationship. Some survivors shared how their partner would tell family members or friends about their use: “We’d be at a family gathering, he would be like nitpickin’ wit’ me, and then, you know, I’m gonna walk away and smoke a cigarette, and he’d be like look at her, she over there smokin’ now.” (S11)

This shame was also used to justify abusive behaviors, as one survivor explained how her partner reacted to her smoking: “That would be like a doorway for him to really unleash a lot of physical and mental and emotional abuse … with the cannabis and the cigarette or tobacco use. It just made it worse because then it’s just more shame, and more hate, and more violence towards me.” (S5)

### Abusive partners enabled undesired substance use and hindered cessation efforts by IPV survivors

Participants shared how abusive partners hindered cessation efforts including providing the substance to the IPV survivor directly or using it in front of them, deliberately causing stress knowing it would encourage substance use, and directly impeding cessation efforts. A survivor shared how her partner tricked her into using cannabis during her pregnancy:

“I didn’t even know that it was an edible until I had took a bite of it ‘cause I felt like my boyfriend was tryin’ to manipulate me. He’s like, ‘Oh, eat this.’ It was like a cookie, and I’m like, ‘Ooh, a cookie.’ You know, you’re pregnant, you just like, ‘Oh, cookie.’ Then I realized what it was, and I’m like, ‘Oh?’ but that was the only thing I had throughout my pregnancy was this edible cookie.” (S6)

An advocate shared about a client whose cessation efforts were impeded: “Well, one person said she was trying to quit, but the baby’s father kept on smokin’ and blowing’ smoke all around her, cigarette and cannabis, just doing it right in front of them, drinking, all of that. There was no respect that person—what that person was trying to do, and even doing it intentionally.” (A12)

IPV survivors described that abusive partners also directly impeded cessation efforts by preventing access to substance use treatment. One survivor detailed how her abusive partner interfered with her ability to use nicotine patches by inserting himself in her obstetrics care decisions:

“He wouldn’t let me go anywhere to do anything because I had said that I wanted to get the [nicotine] patch and stuff … He went to doctor’s appointments with me, and he would say, ‘Oh, she don’t need that. She can just do it on her own.’” (S8)

### Abusive partners use systems-level coercion to harass, threaten, or control perinatal IPV survivors

Many participants shared how IPV and systems-level violence intersected, particularly through the use of CPS and health care interactions. One advocate reflected how control of custody was central to SUC, and how the use of threats served to create a sense of fear in survivors:

“There’s a lot of fear instilled in them by their abusive partner. If you’re using, I could tell CYF [local Child Protective Services agency], and they could take the child away. Or if the abusive partner wants custody of that child, or doesn’t want their partner to seek primary custody, they’ll use CYF as a form of control… ‘I’m gonna tell them you’re using marijuana, and you’re gonna get in trouble if you try to get custody of our child.’” (A8)

This active involvement of CPS was discussed by survivors as well, with one participant reporting how her partner acted on his threats by calling CPS in response to her smoking cigarettes:

“Cause right after I had her [daughter], he called CPS and he told them that I almost dropped her on her head because I fell asleep in a chair with her because I smoked cigarettes. They were like, ‘You can’t fall asleep from a cigarette.’” (S7)

This weaponization of substance use against survivors was also seen in health care settings. Participants described how partners would initiate conversation around substance use during visits to cause shame or embarrassment and to suggest to the health care team that the IPV survivor was not taking appropriate care of the baby:

“I know quite a few of ‘em if their partner even goes with them to the well-baby checkups—’cause the partner will try to use that marijuana use or tobacco use against them saying, ‘Well, she’s supposed to be taking care of this baby’… to use that as something to try to embarrass her or keep her from going to the doctor or being honest with her doctor about that use.” (A14)

Preventing seeking health care was another way that participants described SUC, as abusive partners used the health care system to manipulate and intimidate survivors:

“Your partner may put the fear into you that if you’re seeing a lot of doctors all the time, they’re gonna catch onto the fact that you’re using cannabis or tobacco or something else, so it prevents that victim from seeking prenatal or postnatal care.” (A8)

## Discussion

To the best of our knowledge, this study is the first to examine the perspectives of IPV advocates and survivors around cannabis and nicotine SUC during the perinatal period. Our findings are consistent with research demonstrating links between IPV and cannabis and tobacco use.^11,13,14^ Others have identified SUC manifestations specific to opioid use disorder^30,31^; our study extends this literature to SUC for nicotine and cannabis use, which are more commonly used^17^ and considered more “benign” than opioids.^32,33^ Similar to previous research demonstrating that many women experiencing IPV turn to substances to cope with their trauma symptoms, our participants described using substances to cope with their abuse.^34–37^ Our study builds upon this literature by suggesting the potential functions of different substances in the lives of survivors. Specifically, it highlights how cigarettes are often used as a coping mechanism for stress, while cannabis helps survivors detach or dull the impact of the IPV. Understanding these dynamics is crucial for designing effective cessation interventions that address the underlying needs these substances currently fulfill for survivors as they navigate healing from trauma.

One of the main ways that SUC manifested in this study was by inhibiting cessation efforts for survivors. These findings are significant as IPV survivors are less likely to stop using cannabis or nicotine during pregnancy.^38–40^ Our results also align with previous studies of SUC in opioid use disorder, which showed how partners’ coercive behaviors impacted substance use.^30^ However, cessation looks very different for perinatal opioid use compared to perinatal cannabis and nicotine use. Rather than the clear-cut opioid use disorder guidelines on medication-assisted treatment with opioid agonists for perinatal patients,^41,42^ pharmacotherapy for prenatal tobacco use has insufficient safety and efficacy evidence,^43^ and prenatal cannabis use has no recommended medication options.^43^ Consequently, if patients disclose their nicotine or cannabis use to obstetric providers, they will receive psychosocial interventions, usually brief counseling urging cessation.^44^ Recognition of the ways that cannabis and nicotine SUC affects help-seeking and cessation efforts is necessary to address the complex factors affecting pregnant people’s ability to reduce substance use.

Another key finding was the shame and stigma experienced by perinatal survivors of IPV who used cannabis or nicotine products. Stigma around perinatal substance use perpetuates avoidance of prenatal care and substance use treatment,^45–47^ leading to worse infant health outcomes.^48,49^ Perinatal IPV is also associated with stigma and negative maternal–infant health outcomes such as preterm birth, insufficient prenatal care, and low birth weight.^3,50^ Our study is aligned with prior research and illustrates how experiences of both IPV and perinatal substance use can have compounding effects, preventing pregnant people from living safe, healthy lives. Furthermore, our results demonstrate how shame based on perinatal survivors’ substance use is often used as a tool for emotional abuse and manipulation. Given these findings, it is crucial to enhance health care providers’ awareness of intersectional stigma through training programs, as well as to develop programs for systems (such as health care) to combat stigma experienced by perinatal IPV survivors with substance use disorders.

Participants described how SUC manifested through the abusive partner’s involvement of systems, particularly threatening potential involvement of CPS. Past literature has shown that CPS involvement can be harmful for IPV survivors. One study evaluated IPV survivors’ perspectives on mandated reporting for IPV; half said the report made their situation much worse due to involvement with the criminal legal system and removal of their children.^51^ Past work found that CPS involvement and child custody were commonly used by partners as a way to threaten or coerce perinatal people using opioids.^30,52,53^ Our study expands the current literature by showing how fear of CPS is a form of SUC in the perinatal period that extends beyond illicit or illegal drugs to include more common and legal substances such as cannabis and nicotine. Addressing this fear is important for systems supporting people in the perinatal period, a time where there is particular concern around substance use detection and connection with help-seeking resources due to potential consequences from CPS.^54^ Results from this study also suggest how coercion can be multilevel and include both individual (*e.g.*, SUC) and structural violence (*e.g.*, CPS involvement).

Health care is an important space to provide support around perinatal cannabis and nicotine use. However, despite nicotine and cannabis being legal in many states and current health care guidelines encouraging open discussion of use with patients,^55^ these conversations do not always occur. This gap has been attributed to various factors, including patients’ fear around disclosing their use, health care providers not initiating the conversation, and patients’ experience of stigma.^56–58^ Our findings offer an additional explanation for the hesitancy to disclose, as participants emphasized how abusive partners instill fear in survivors when interacting with providers and obstruct access to adequate perinatal care. The coercive power exerted by abusive partners is likely amplified by the shame and stigma surrounding perinatal substance use, underscoring the urgent need for effective, scalable, survivor-centered health care policies and interventions that focus on reducing shame and connecting survivors to resources.

There are limitations to the study worth noting. This study took place in the Midwest, and results may not be applicable to other areas, as states have different policies regarding cannabis legality, mandated reporting, and involvement of CPS during the perinatal period. Furthermore, using convenience sampling, including a research recruitment repository, may have led to selection bias, as the narratives and backgrounds of those who are interested in research may differ from those who do not feel comfortable engaging in research. We also were unable to include people who use languages other than English, and none of the survivors identified as gender diverse. People who use languages other than English and those who are gender diverse are uniquely impacted by IPV,^59,60^ and their perspectives should be included in future studies. Interviews with a sample recruited from a different region or setting might generate different themes. It is important to note, however, that the intention of qualitative research is not to achieve generalizability but rather to understand people’s rich stories and lived experiences.^26^ Finally, to minimize stigma and ensure participants felt comfortable, we did not ask participants about the frequency and quantity of substance use. This information will be helpful in future studies to better understand the patterns and impacts of SUC among perinatal IPV survivors.

Despite the limitations, this work lays a foundation for future research as well as practice and policy innovation. Longitudinal studies exploring how cannabis and nicotine SUC manifests throughout the perinatal period are needed to better design interventions and supports that meet the unique needs of this population. Future studies should also consider the perspectives of CPS caseworkers, family law experts, and family members of IPV survivors, all of whom may interact with and support perinatal survivors of nicotine and cannabis SUC. Interviewing other invested parties will be helpful in creating more education geared toward reducing stigma and improving perinatal IPV survivors’ experiences engaging with settings that are intended to be supportive.

Findings also highlight key areas for systems-level practice and policy transformation. Multilevel interventions around nicotine and cannabis, which are cocreated by IPV survivors, are needed for people and systems who serve perinatal people, including CPS, health care, substance use facilities, family law, and law enforcement. Recent literature on systems-level IPV responses recommends greater integration of health and advocacy service delivery through clinician training with an emphasis on a trauma-informed approach, electronic health record tools, warm referrals to victim services agencies or colocated IPV advocates (*e.g.*, IPV advocates working at hospitals or CPS offices), and cross-sector partnerships.^16,61,62^ Current guidelines for evidence-based, systems-level treatment of substance use disorders similarly emphasize an integrated, colocalized approach that includes case management, peer support, and trauma-informed behavioral health services.^63,64^ Because SUC is pervasive and there are multiple barriers to disclosure, we argue that systems shift away from universal IPV screening, toward the use of universal empowerment approaches, where *all* patients receiving care from specific systems (*e.g.*, during prenatal or newborn visits for health care systems) are given education and resources around IPV.^65,66^ Universal empowerment approaches would ensure that resources reach all patients without the need to self-identify as an IPV survivor, empowering perinatal people to seek help when they are ready or support others who are experiencing IPV.

Trainings around SUC should also be developed and widely implemented for all systems that work with perinatal IPV survivors or abusive partners. All systems serving perinatal IPV survivors should have direct connection to victim services agencies, to ensure easy connection to housing, legal, mental health, and harm reduction services, as previous research has recommended a multidisciplinary and comprehensive approach.^67^ Any systems-level policy around perinatal substance use (*e.g.*, urine substance use screening at labor and delivery, policies around mandated reporting) must consider the potential influences of SUC and the way nicotine and cannabis use may be weaponized by abusive partners. All interventions, trainings, and policies should be developed in collaboration with IPV survivors and advocates, to ensure solutions are survivor-centered and highly impactful. Our findings indicate the importance of transformative solutions that prioritize healing, social support, and resource connection for perinatal IPV survivors experiencing cannabis and nicotine SUC.

## Supplementary Material

Supplementary Data S1

Supplementary Data S2

## Abbreviations Used

IPV: Intimate partner violence
SUC: Substance use coercion
