# Supplementary material for: Cannabis and Nicotine Substance Use Coercion During the Perinatal Period
Source: Womens Health Rep (New Rochelle). 2025 Oct 8;6(1):1153–62. doi: 10.1177/26884844251387024 (PMC12549175; doi:10.1177/26884844251387024)
Supplement: Supplementary Data S1 [file 26884844251387024_supplementary_data_s1.docx]

**Substance Use Coercion IPV Advocate Interview Guide**

**Review information script**

Are you somewhere that you feel safe to talk? Are you somewhere that is private, away from others?

**Do we have your permission to record this interview/meeting?** All recordings will be kept in a secure and confidential location and will not be linked to any of your identifiable information such as name or contact.

Throughout the course of the interview, we would love to hear stories from your work. Any identifying information from stories will be removed and any stories you share will be completely anonymous.

**To start, please tell me about the work that you do.**

- Briefly, what role do you play in your clients’ journey?
- What are your interactions with clients/patients usually like?

**Tell me about the challenges and stressors of IPV survivors who are pregnant or recently postpartum?**

- Are there any additional considerations you take in your work when supporting pregnant and postpartum survivors of IPV?

**Have you ever seen or known any pregnant clients to be smoking cigarettes or tobacco?**

- If Yes: How was this handled by your organization?
- Did you talk with them about their smoking at all? Why or why not? How did that conversation go?
- How did their smoking relate to their abuse and/or their abuser?

**Have you ever seen or know any pregnant clients to be using weed or cannabis?**

- If Yes: How was this handled by your organization?
- Did you talk with them about their weed/cannabis use at all? Why or why not? How did that conversation go?
- How did their weed/cannabis relate to their abuse and/or their abuser?

**Tell me about, in general, the challenges and stressors of pregnant and recently postpartum IPV survivors who use substances, particularly cannabis/marijuana and tobacco.**

- Tell me about the factors that increase the likelihood of a pregnant and postpartum IPV survivor may use cannabis or tobacco?
- How may cannabis or tobacco be used to cope with violence and trauma?

**Now, I am curious about your thoughts about how IPV and tobacco and cannabis use may be related, especially for people who are pregnant or post partum.**

**Tell me a little bit about how an abusive partner may influence the IPV survivors use of cannabis and tobacco?**

**Sometimes people use the word “substance use coercion” to talk about when abusers try to use their partner’s substance use to control, manipulate, or hurt them.**

- Tell me how you have seen this show up in your work.
- Have you seen this in terms of survivors’ tobacco and cannabis use?
- How has this shown up? In what ways?
- How may people who use violence (abusive partners) hinder someone’s ability to quit tobacco or cannabis, particularly during their pregnancy?
- **Are there any survivor stories that come to mind when you are responding to this question?**
- **Not provide identifying information, but share stories of survivors if you can, we will remove identifying information.**

**Tell me about the supports and services available for pregnant or recently postpartum IPV survivors experiencing coercion around tobacco and cannabis. Tell me about the supports and services you wish were available?**

**One of our goals is to help healthcare providers be more supportive and helpful to pregnant people who have experienced relationship stress and cannabis/tobacco use. Tell me about your thoughts about this.**

**Thank you so much for completing this interview. Is there anything else I should have asked or anything you would like to share?**
