# Supplementary material for: Cannabis and Nicotine Substance Use Coercion During the Perinatal Period
Source: Womens Health Rep (New Rochelle). 2025 Oct 8;6(1):1153–62. doi: 10.1177/26884844251387024 (PMC12549175; doi:10.1177/26884844251387024)
Supplement: Supplementary Data S2 [file 26884844251387024_supplementary_data_s2.docx]

**Interview Guide: IPV Survivor**

**Are you somewhere that you feel safe to talk? Are you somewhere that is private, away from others?**

**Do we have your permission to record this interview/meeting?** All recordings will be kept in a secure and confidential location and will not be linked to any of your identifiable information such as name or contact.

**Tell us a little about your pregnancy. What was the most joyful/challenging part?**

**Now we are going to transition to talking about tobacco and cannabis use during the pregnancy and postpartum period. Did you use any tobacco during your pregnancy, like cigarettes?**

- What role did tobacco play in your life at the time (how important was it, how often did you use it, why did you use it)? How did it affect your pregnancy?
- Did you continue/start smoking again after your pregnancy?

**Did you use any cannabis or weed during your pregnancy, through smoking, vaping, or edibles?**

- What role did cannabis/weed play in your life at the time (how important was it, how often did you use it, why did you use it)? How did id affect your pregnancy?
- Did you continue/start using again after your pregnancy?

**What did your partner think about your tobacco and/or cannabis use?**

- How did this change with pregnancy or after your pregnancy?
- How did your partner influence your use of these substances?
- How did your partner’s thoughts or opinions about your tobacco and/or cannabis use relate to their controlling or abusive behaviors?

**What role did substances play in your relationship, if any?**

- How did your use of tobacco or cannabis relate to the stress or violence in your relationship?
- How did your use of tobacco or cannabis relate to your experience of being pregnant or postpartum while in an abusive relationship?
- How did your partner try and control your use of tobacco or cannabis during or after your pregnancy?

**Sometimes people talk about the ways their partners tried to use substances like cannabis or tobacco to control them or their lives or hurt them. Tell me about how your partners may have used substances to control your life or hurt you?**

**How do you think controlling behaviors around cannabis and tobacco use are similar? How are they different?**

**Tell us about your thoughts about quitting tobacco or cannabis use during pregnancy.**

- Tell us about what your partner thought or did about you stopping tobacco or cannabis use.
- Did your partner ever hinder your efforts to seek help? If yes, how?

**Tell us about your interactions with law enforcement, child protective services, healthcare, or other systems regarding your tobacco and cannabis use.**

- How was the process?
- How did your partner play a role with that?
- In what ways did these systems help you, and in what ways may they have harmed you?

**One of our goals is to help healthcare providers be more supportive and helpful to pregnant people who have experienced relationship stress and cannabis/tobacco use. Could you tell me about your thoughts about this?**

- What do you wish healthcare providers knew about the experience of people at the intersection of pregnancy, intimate partner violence, and cannabis or tobacco use?
- Were there any resources or interactions that you found helpful?
- If in shelter, did your substance use change during your time at the shelter? What resources did the shelter connect you with (or not)?
- What resources do you wish were available for people experiencing pregnancy and intimate partner violence who use cannabis or tobacco?
